# Pseudo-Wellens syndrome from sepsis-induced cardiomyopathy: a case report and review of the literature

**DOI:** 10.1186/s13256-021-02756-y

**Published:** 2021-04-06

**Authors:** Teressa Reanne Ju, Ilhwan Yeo, Gregory Pontone, Reema Bhatt

**Affiliations:** 1 Department of Internal Medicine, NewYork-Presbyterian Queens, Flushing, USA; 2 Department of Cardiology, NewYork-Presbyterian Queens, Flushing, USA; 3 Department of Cardiology, Weill Cornell Medicine, New York, USA

**Keywords:** Wellens syndrome, Pseudo-Wellens syndrome, Coronary artery disease, Heart failure, Sepsis

## Abstract

**Background:**

Pseudo-Wellens syndrome is a rare entity characterized by the presence of electrocardiogram (ECG) changes of Wellens syndrome but without the stenosis of the left anterior descending (LAD) coronary artery. In previous reports, pseudo-Wellens syndrome most commonly resulted from recreational drug use or unidentified etiologies. We present a unique case of pseudo-Wellens syndrome due to sepsis-induced cardiomyopathy and a review of the literature.

**Case presentation:**

A 62-year-old Caucasian woman was admitted for sepsis from left foot cellulitis. Laboratory data were notable for elevated lactate of 2.5 mmol/L and evidence of acute kidney injury. She developed chest pain on the third day of hospitalization. ECG showed symmetric T-wave inversion in leads V1–V4. Serial troponin I levels were within normal limits. Chest imaging showed no pulmonary embolism. Echocardiogram showed ejection fraction of 25%, left ventricular diastolic diameter of 4.6 cm, and multiple segmental wall motion abnormalities. Cardiac catheterization showed patent coronary arteries. The hospital course was complicated by transient sinus bradycardia and hypotension. She was hospitalized for a total of 17 days. ECG prior to discharge showed resolution of T-wave changes.

**Conclusion:**

Pseudo-Wellens syndrome may result from myocardial ischemia due to vasospasm or myocardial edema from external insults. In our case, we suspect sepsis-related cytokine production resulting in cardiomyopathy and pseudo-Wellens syndrome. The clinical manifestations were indistinguishable between Wellens and pseudo-Wellens syndrome. Physicians should include the diagnosis of pseudo-Wellens syndrome when considering the presence of LAD coronary artery occlusion given risk stratifications.

## Background

Wellens syndrome is an electrocardiographic (ECG) pattern of T-wave changes that indicates critical stenosis of the left anterior descending (LAD) coronary artery and warrants urgent intervention. Several conditions can mimic Wellens syndrome, such as cocaine use [1], marijuana use [2], myocardial bridging [3], and pulmonary embolism [4]. We present a case of pseudo-Wellens syndrome secondary to sepsis. In addition, we summarize the clinical presentations of 21 reported cases and outline the commonality of pseudo-Wellens syndrome.

## Case presentation

A 62-year-old Caucasian woman with a history of stroke, epilepsy, and peptic ulcer disease presented to our emergency department (ED) for increasing swelling and erythema over her left foot despite 7 days of oral antibiotic therapy for cellulitis. She denied fever, chills, and respiratory or gastrointestinal symptoms. She had no family history of cardiac diseases, nor did she have any previous cardiac workup or echocardiograms. She was an active smoker with a 50-pack year smoking history and denied alcohol or drug use. Physical examination upon admission was pertinent for erythema, swelling, and tenderness in the left lower extremity. Neurologic examination was intact except for left-sided hemiparesis from a previous stroke. Laboratory tests were notable for lactate of 2.5 mmol/L. Her serum creatinine increased from 0.6 to 0.92 mg/dL within 24 hours upon admission, consistent with a diagnosis of acute kidney injury. She was hospitalized under the diagnosis of cellulitis complicating sepsis with end-organ dysfunction and started on intravenous vancomycin 1000 mg daily.

She was clinically stable until the third day of hospitalization, when she began to have intermittent episodes of hypoxia with a measured oxygen saturation of 88%, along with substernal chest discomfort. Physical examination was pertinent for diffuse wheezing in all lung fields which was alleviated after receiving nebulized albuterol. The following day, hypotension was noted, with a systolic blood pressure of 75 mmHg that resolved after fluid resuscitation. Her electrocardiogram (ECG) at the time showed sinus tachycardia. Serial troponin I levels were within normal limits. Computed tomography angiogram of the chest was negative for pulmonary embolism. Echocardiogram revealed an ejection fraction of 25%, left ventricular diastolic dimension of 4.6 cm, and multiple segmental wall motion abnormalities in the basal-to-mid anteroseptum along with basal-to-mid anterior, apical anterior, and apical septum.

**Fig. 1:**
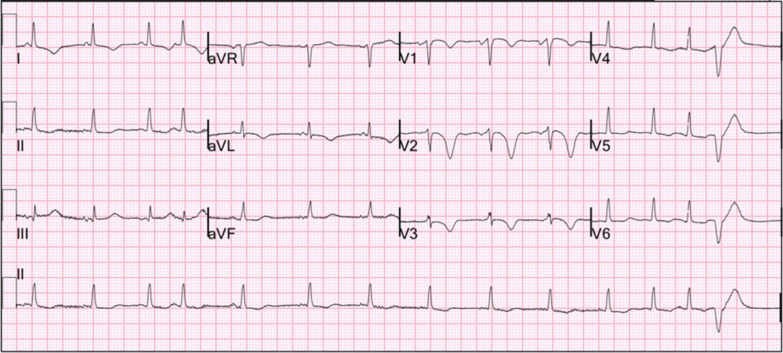
Electrocardiogram while patient had chest pain: sinus arrhythmia with deep symmetric T-wave inversion in precordial leads V1–V4 consistent with Wellens syndrome

**Fig. 2:**
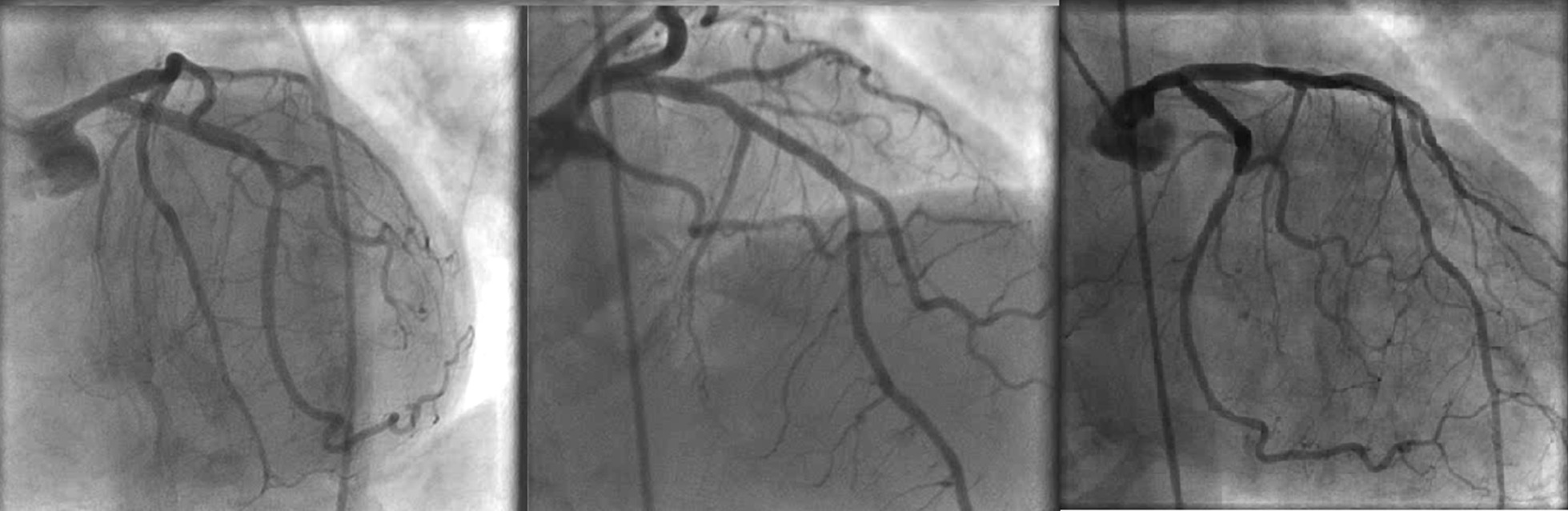
Cardiac catheterization: patent coronary arteries with mild irregularities in both left anterior oblique-caudal projection (left) and right anterior oblique-cranial projection (middle). The right anterior oblique-caudal projection (right) shows a patent proximal left anterior descending coronary artery

**Fig. 3:**
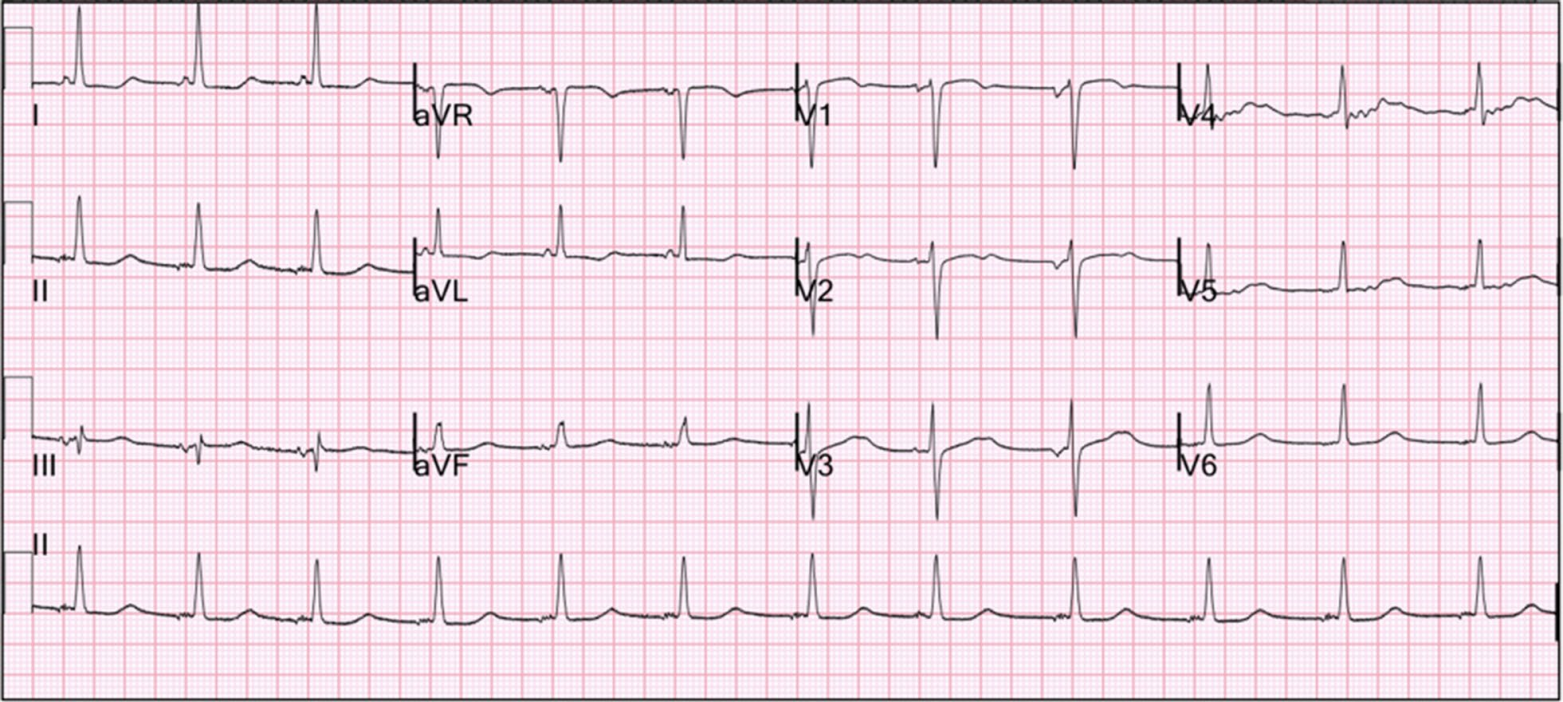
Electrocardiogram prior to discharge: disappearance of the T-wave inversions after resolution of chest pain

Repeat ECG 8 hours after the initial one was remarkable for sinus arrhythmia with deep symmetric T-wave inversions in leads V1–V4, consistent with Wellens syndrome (Fig. 1). Emergency cardiac catheterization revealed patent coronary arteries without obstructive coronary artery disease (CAD) (Fig. 2). Following her procedure, she was admitted to a cardiovascular intensive care unit due to alternating episodes of sinus bradycardia and tachycardia accompanied by hypotension which resolved spontaneously without medical management. Her chest pain resolved after day 6 of cardiac catheterization. After 17 days of hospitalization, she was discharged with metoprolol extended-release 25 mg daily, atorvastatin 80 mg daily, and furosemide 20 mg daily. An ECG prior to discharge showed normal sinus rhythm without T-wave abnormalities (Fig. 3). An appointment was made to follow up with our cardiology clinic and a repeat echocardiogram was scheduled. However, she was lost to follow-up, and further attempts to reach the patient by phone were unsuccessful.

## Discussion

The ECG pattern of Wellens syndrome was first described by Gerson *et al*. in 1979 [5], who noted ECG findings of exercise-induced inverted terminal T waves in the precordial leads in patients with proximal LAD ischemia. In 1982, de Zwaan and Wellens reported a case series of 145 patients with unstable angina. Among these patients, 26 (18%) had similar ECG findings: ST–T segment in leads V2 and V3 consisting of an isoelectric or minimally elevated (1 mm) takeoff of the ST segment from the QRS complex, a concave or straight ST segment passing into a negative T wave at an angle of 60 to 90°, and a symmetrically inverted T wave [6]. In a subsequent prospective study which assessed patients who were admitted due to unstable angina, de Zwaan revealed that 128 of 1260 patients with similar ECG findings all had critical narrowing of the proximal LAD coronary artery [7]. As of today, the criteria for Wellens syndrome are as follows: history of anginal chest pain, minimal or no elevation of cardiac enzymes, no significant ST segment elevation (< 1 mm), no pathological precordial Q waves, no loss of precordial R-wave progression, and deeply inverted or biphasic T waves mainly in leads V2 and V3 and sometimes in leads V1, V4, V5, and V6 [8, 9]. Wellens syndrome has since been used to identify critical proximal LAD coronary artery stenosis in patients with unstable angina [8].

Pseudo-Wellens syndrome is a term used to describe a constellation of clinical presentations and ECG pattern similar to Wellens syndrome but without the finding of critical stenosis of LAD coronary artery. Table 1 summarizes 22 reported cases of pseudo-Wellens syndrome [1–4, 10–22]. The average age was 50 years (range 22–81), and four patients (18.1%) were female. In six of these cases, illicit drug use such as cocaine and phencyclidine were identified. Five cases had no identified causes. The majority of cases had presenting symptoms of chest pain, while the duration of symptoms varied from a few hours to a few months. Normal to mildly elevated serum cardiac enzymes were noted. Resolution of ECG changes occurred when chest pain resolved in some but not all cases. Coronary angiography was mostly unremarkable. In the absence of existing CAD, pseudo-Wellens syndrome had a favorable prognosis. There was no mortality reported in the cohort.

**Table 1:** Reported cases of pseudo-Wellens syndrome in literature

Article	Etiology	Age, gender	Chief complaint, duration	Reported time of ECG change resolution	Peak troponin I level (ng/ml)	Angiography finding
Langston *et al*. 2006 [10]	Cocaine	46, M	Chest pain, 45 minutes	48 hours	Normal	Normal
Batra *et al*. 2008 [11]	Injection drug use	41, F	Chest pain, several weeks	Not reported	0.04	Normal
Dhawan *et al*. 2008 [1]	Cocaine	41, M	Chest pain, 3 hours	24 hours	Normal	Normal
Bucciarelli-Ducci *et al*. 2007 [12]	Unknown	73, F	SOB, not reported	Persistent at 17 days	1.1	80% stenosis in RCA
Bucciarelli-Ducci *et al*. 2007 [12]	Unknown	41, M	Asymptomatic (found during routine visit)	Persistent at 30 days	Normal	20% stenosis in the mid-LAD coronary artery 20% stenosis in the mid-RCA
Bucciarelli-Ducci *et al*. 2007 [12]	Unknown	45, M	Chest pain, not reported	30 days	Normal	Normal
Migliore *et al*. 2011 [13]	Myocardial bridge	78, M	Chest pain, not reported	6 weeks	3.05	Myocardial bridge in LAD coronary artery
Migliore *et al*. 2011 [13]	Takotsubo syndrome	62, F	Chest pain, not reported	6 weeks	2.3	Normal
Migliore *et al*. 2011 [13]	Acute cholecystitis	81, F	Chest pain, not reported	Persistent for 7 days, resolved after 6 weeks	2.01	Normal
Abulaiti *et al*. 2013 [14]	Unknown	47, M	Chest pain, 2 months	When chest pain resolved	Normal	50% stenosis in proximal LAD coronary artery
Oksuz *et al*. 2015 [15]	Unknown	33, M	Chest pain, 1 hour	When chest pain resolved with nitroglycerin	Normal	Normal
Co *et al*. 2017 [2]	Marijuana	22, M	SOB, 5 days	7 months	Normal	Angiography is not performed
Kaplanis *et al*. 2017 [3]	Myocardial bridge	55, M	Chest pain, 3 days	Not reported	Normal	Myocardial bridge in the mid-LAD coronary artery
Lin *et al*. 2017 [16]	Cocaine	52, M	Chest pain, 1 day	When chest pain resolved	Normal	Non-occlusive LAD coronary artery
Inayat *et al*. 2018 [17]	Cannabis, PCP	41, M	Chest pain, 6 hours	12 weeks	Normal	Normal
Kumar *et al*. 2018 [18]	Cocaine	27, M	Chest pain, not reported	1 week	Not reported	Not performed
Sedhai *et al*. 2018 [4]	Pulmonary embolism	22, M	Chest pain, not reported	Not reported	Normal	Not performed
Grautoff *et al*. 2019 [19]	Acute cholecystitis	54, M	Epigastric pain, not reported	Not reported	Not reported	Not performed
Muhailan and Al-Shbool 2019 [20]	Nivolumab/ipilimumab	70, M	Syncope, not reported	2 weeks	1.94	Normal
Ola and Tak 2019 [21]	HTN and LVH	61, M	Chest pain, 3 days	When hypertension resolved	Normal	Normal
Effoe *et al*. 2019 [22]	Acute pancreatitis	45, M	Chest pain,	When chest pain resolved (24 hours)	Normal	Anomalous origin of the dominant RCA from the opposite sinus
Ju *et al*. 2020 (present case)	Sepsis	62, M	Chest pain, one day	6 days	Normal	Normal

*PCP* phencyclidine, *HTN* hypertension, *LVH* left ventricular hypertrophy, *M* male, *F* female, *SOB* shortness of breath, *RCA* right coronary artery, *LAD* left anterior descending

The exact mechanism of ECG changes in pseudo-Wellens syndrome is unclear. Historically, ECG findings were explained by transient impedance of coronary flow leading to myocardial ischemia [10, 15]. As vasospasm resolved, ECG changes recovered to baseline and symptoms resolved. In another study utilizing contrast-enhanced cardiac magnetic resonance imaging, Migliore *et al*. found myocardial edema, rather than ischemia, underlying the Wellens ECG pattern [13]. The ECG pattern was persistently present until myocardial edema resolved. In our case, we postulate myocardial edema as a result of sepsis-related cytokine production, resulting in cardiomyopathy and pseudo-Wellens syndrome [23].

Takotsubo cardiomyopathy and sepsis-induced cardiomyopathy may be difficult to differentiate clinically. However, some differences exist in the pathophysiology and echocardiographic findings between the two entities [24]. Takotsubo cardiomyopathy typically leads to regional wall dysfunction, mostly described as apical ballooning of the distal ventricle with hyperkinesis of the basal walls secondary to catecholamine surge [25]. However, two anatomical variants, hypokinesis of the mid-ventricular segments only and hypokinesis of the basal segments only, account for 15% and less than 5% of cases with Takotsubo cardiomyopathy, respectively [26]. The Mayo Clinic criteria for diagnosing Takotsubo cardiomyopathy includes three key components: regional wall motion abnormalities extending beyond a single epicardial vascular distribution; absence of obstructive coronary disease; and presence of electrocardiographic abnormalities [27]. On the other hand, in sepsis-induced cardiomyopathy, the cytokine storm leads to myocardial dysfunction, perhaps caused by mitochondrial dysfunction. This may be displayed as various echocardiographic findings, such as global or regional wall motion abnormalities during systole and/or diastole [24, 28]. The regional wall motion abnormalities in our patient’s echocardiograms were located in the territory of the LAD coronary artery. Therefore, the image findings are inconsistent with the Mayo Clinic definition of Takotsubo cardiomyopathy and more in favor of sepsis-induced cardiomyopathy. Nevertheless, as supportive therapy represents the mainstay of treatment for the sepsis-induced and Takotsubo cardiomyopathies, the distinction in diagnosis should not impact the overall clinical management.

## Conclusion

Pseudo-Wellens syndrome is a rare entity which mimics Wellens syndrome but without the presence of LAD coronary artery stenosis. It likely results from transient myocardial ischemia secondary to vasospasm or myocardial edema due to external insults. While underlying CAD cannot be ruled out based on clinical presentations, physicians should be vigilant to identify ECG pattern of Wellens syndrome and consider early cardiac catheterization to rule out LAD coronary artery pathology.

## Data Availability

Not applicable.

## Abbreviations

LAD: Left anterior descending
ECG: Electrocardiogram
CAD: Coronary artery disease
